# A Married Man’s First Duty

**Published:** 1892-07

**Authors:** Mary E. Spencer


### A MARRIED MAN'S FIRST DUTY.

When a young man marries his first duty is to study how his wife may live most beautifully and most honorably, and for the longest number of years. Our homes ought to anticipate permanence and have the common sense and morals that win long continuance. There can be no happiness like that of a noble home, no misery like that of a merely coupling together of man and woman for a brief hard working and animal existence. Every day should be lived so as to add health, rather than to enfeeble and weary. You may be sure that if you cannot rise in the morning with a clear head and pure heart, there is little before you but disease and misery and discord.—MARY E. SPENCER in *St. Louis Globe-Democrat.*

